# Effects of Ten Sessions of High Frequency Repetitive Transcranial Magnetic Stimulation (HF-rTMS) Add-on Treatment on Impulsivity in Alcohol Use Disorder

**DOI:** 10.3389/fnins.2019.01257

**Published:** 2019-12-04

**Authors:** Renée S. Schluter, Ruth J. van Holst, Anna E. Goudriaan

**Affiliations:** ^1^Department of Psychiatry, Amsterdam Institute for Addiction Research, Amsterdam UMC, University of Amsterdam, Amsterdam, Netherlands; ^2^Department of Research and Quality of Care, Arkin, Amsterdam, Netherlands; ^3^Jellinek, Amsterdam, Netherlands

**Keywords:** alcohol use disorder, alcohol dependence, transcranial magnetic stimulation, impulsivity, delay discounting, go-nogo, stop signal, neuromodulation

## Abstract

**Introduction:**

Alcohol use disorder (AUD) is characterized by increased impulsivity, which is multifactorial and can be assessed by tests like the delay discounting, Go-Nogo, and stop signal task (SST). Impulsivity has been related to poor treatment outcomes in substance use disorders, including AUD. In order to decrease impulsivity or improve inhibitory control, high frequency transcranial magnetic stimulation (HF-rTMS) has gained interest. Studies applying HF-rTMS over the DLPFC of individuals suffering from AUD assessing its effects on impulsivity measures are scarce, and results are inconclusive.

**Methods:**

The current study (registered in Netherlands Trial Register with trial number 5291: https://www.trialregister.nl/trial/5151) applied 10 sessions of HF-rTMS [sixty 10 Hz trains of 5 s at 110% motor threshold (MT)] over the right DLPFC of 80 alcohol dependent patients in clinical treatment on 10 consecutive workdays. At baseline, halfway and after the HF-rTMS treatment, the delay discounting, Go-NoGo, and SST were assessed.

**Results:**

Ten sessions of HF-rTMS over the right DLPFC versus sham HF-rTMS did not affect performance on the delay discounting, Go-NoGo, and SSTs. A significant effect of age was found for the Go-NoGo task, with higher age associated with better performance. Furthermore, no significant correlations were found between difference scores of task performance and baseline impulsivity or severity of AUD.

**Discussion:**

Results of this study, in combination with other studies using HF-rTMS studies in alcohol and substance use disorder, indicate mixed and inconclusive findings of HF-rTMS on impulsivity. Future studies within patient groups hospitalized at the same department are recommended to consider using a sham coil that mimics the sensations on the scalp of active HF-rTMS and to measure motivation across test sessions.

## Introduction

Worldwide approximately 2.6% of the population is suffering from alcohol use disorder (AUD) (World Health Organization, 2018). AUD is characterized by loss of control over alcohol intake despite awareness of the negative social, health, and financial consequences (Baler and Volkow, 2006). The loss of control over intake is caused by decreased inhibitory control capacities observed in AUD (Lawrence et al., 2009). From a neurobiological perspective, this has been associated with diminished functioning of the prefrontal cortex (Koob and Volkow, 2010).

Impulsive behavior can be defined as acting without foresight or careful deliberation (Dalley and Robbins, 2017) and therefore can result in unduly risky or inappropriate behavior, often with undesirable consequences (Dawe et al., 2004; Lee et al., 2019). Impulsivity has been related to poor treatment outcomes in substance and AUD (Goudriaan et al., 2011; Stevens et al., 2014; Loree et al., 2015; Moeller et al., 2016). Impulsivity is a multifaceted construct which can be subdivided into *delayed reward (choice) impulsivity* and *rapid response impulsivity* (Hamilton et al., 2015a). The former reflects the preference for immediate reward in favor of a larger later reward. The latter reflects the tendency toward immediate action, which is often incompatible with present demands of the situation. Within the rapid response impulsivity construct, two more types can be dissociated, namely *failure to refrain from action initiation* and *inability to stop an initiated response* (Hamilton et al., 2015a). These different constructs of impulsivity can be assessed using different computerized tasks. Choice impulsivity can be assessed using the delay discounting task (DDT) (Bickel and Marsch, 2001). In this task, the more often a participant chooses the lower immediate reward, the more the subjective value of the larger later reward reduces over time, and the more impulsive an individual is considered to be (Odum, 2011; Hamilton et al., 2015b). The Go-NoGo Task (GNGT) assesses the failure to refrain from action initiation. In this task, the more often a participant responds to a stimulus when no response was required (i.e., a false alarm), the more impulsive the individual is considered to be (Hamilton et al., 2015a). The inability to stop an initiated response can be assessed by using the stop signal task (SST) (Verbruggen and Logan, 2008). The task determines the time a participant needs between the go-signal and the stop-signal in order to be able to stop the initiated response in 50% of the time [i.e., the stop signal reaction time (SSRT)]. The higher the SSRT, the more impulsive an individual is considered to be (Hamilton et al., 2015a). Taken together, previous research has shown that individuals with substance use disorders (including alcohol) show impaired performance on the above described impulsivity tasks (Lipszyc and Schachar, 2010; Smith et al., 2014; Wright et al., 2014; Amlung et al., 2017; Sion et al., 2017).

In order to decrease impulsivity, or improve inhibitory control, Transcranial Magnetic Stimulation (TMS) has gained interest (Bellamoli et al., 2014). With TMS, a strong magnetic pulse, originating from an electromagnetic coil, penetrates the skull and changes neuronal activity in the underlying tissue. When pulses are repetitively applied in trains, it is referred to as repetitive TMS (rTMS). Depending on the stimulation frequency this can either be inhibitory (low frequency; LF) or excitatory (high frequency; HF) (Rossi et al., 2009; Guse et al., 2010). A target area frequently chosen within the inhibitory control network, consisting of the prefrontal cortex (Ridderinkhof et al., 2004), is the dorsolateral prefrontal cortex (DLPFC). Results from studies applying HF-rTMS over the DLPFC in substance (including alcohol) dependence show inconsistent effects on impulsivity measures. While one single session of 10 Hz did not improve accuracy on the GNGT (Herremans et al., 2013), four sessions of 10 Hz stimulation did increase accuracy on the GNGT (Del Felice et al., 2016) in alcohol dependent patients. In nicotine dependence, one single session of 10 or 20 Hz stimulation improved performance of the DDT (i.e., less discounting for monetary as well as cigarette rewards) (Sheffer et al., 2013), suggesting decreased impulsivity. So far, no studies tested the effect of HF-rTMS in alcohol dependence on DDT and SST performance.

In the current study, the effect of 10 HF-rTMS sessions on impulsivity in individuals in treatment for AUD is investigated. We hypothesized that 10 HF-rTMS sessions would improve impulse control abilities. We, therefore, expected to find that after active treatment compared to sham treatment, impulsivity would be decreased. Eighty AUD individuals were included in the study and treated with either 10 active or 10 sham HF-rTMS sessions on 10 consecutive workdays added on to their treatment as usual. Impulsivity tasks were assessed before, in between, and after the HF-rTMS treatment.

## Methods

### Study Design

The effect of the HF-rTMS add-on treatment on impulsivity was studied in a parallel, single center, single blind trial in abstinent alcohol dependent subjects, randomized (1:1) to either treatment as usual (TAU) plus 10 sessions of active HF-rTMS or TAU plus 10 sessions of sham HF-rTMS, as described elsewhere in detail (Schluter et al., 2018). This study was approved by the Medical Ethical Committee of the Academic Medical Centre Amsterdam (2015_064) and is registered in The Netherlands Trial Register (NTR) with trial number 5291. Informed consent of all participants was obtained after explanation of all study procedures and before screening for the in- and exclusion criteria.

### Study Sample

All participants were recruited at an addiction treatment centre in Amsterdam (Jellinek, Amsterdam, The Netherlands), and were abstinent during participation to the current study. Here they received 6 weeks of a fulltime treatment program of Cognitive Behavioral Therapy (CBT) or Acceptance and Commitment Therapy (ACT) supplemented with emotion regulation training and motivational enhancement therapy. Besides these group sessions, every participant had individual sessions with a psychologist and a mentor every week. In the session with the psychologist, comorbidities, and other problems of the patients that occurred during treatment were discussed. During the mentor sessions supportive CBT or ACT focusing on remaining abstinent were given. Finally, some of the patients received pharmacotherapy. Inclusion criteria were a recent DSM-IV diagnosis of alcohol dependence (i.e., less than 4 months after detoxification) and an age between 20 and 65. Exclusion criteria were (1) insufficient knowledge of the Dutch language, (2) Montreal Cognitive Assessment (MOCA) score below 10, (3) current DSM-IV diagnosis of depression, schizophrenia or another psychotic disorder, (4) current recreational drug use, and (5) HF-rTMS contraindications [such as a history of epileptic seizures, metal implants near the head or use of the following medication: imipramine, amitriptyline, doxepine, nortriptyline, maprotiline, chlorpromazine, clozapine, foscarnet, ganciclovir, ritonavir, theophylline (Rossi et al., 2009)].

### Procedure

**FIGURE 1:**
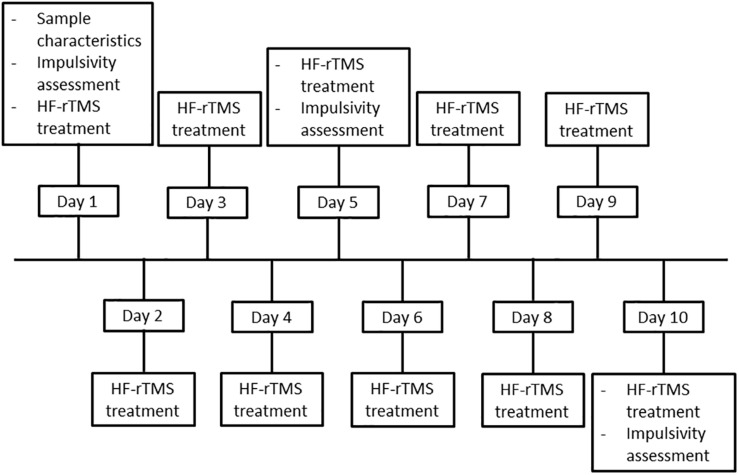
Flowchart of the study procedure.

When an individual met all inclusion and no exclusion criteria, he or she was enrolled in the study. In order to assure concealed randomization, participants were randomized into the sham or active stimulation group, based on the stratification factors anti-craving medication (yes/no) and age (20–40/41–65) using the randomization module implemented in the data management system Castor EDC (Castor Electronic Data Capture, Ciwit BV, Amsterdam, Netherlands, 2016). After randomization, participants started with the research procedure, which took place on 10 consecutive workdays. During the first session (baseline), sample characteristics were assessed, after which the impulsivity tasks were performed. Subsequently, stimulation intensity and location were determined, and the first HF-rTMS treatment was delivered. During the second to fourth session, HF-rTMS treatment was delivered. During the fifth session, HF-rTMS treatment was performed, followed by assessment of the impulsivity tasks. The sixth to ninth session only contained HF-rTMS treatment. The 10th session was identical to the fifth session. For an overview of the procedure, see Figure 1.

### Intervention

The active intervention existed of 10 HF-rTMS (sixty 10 Hz trains of 5 s at 110% MT treatment sessions over the right DLPFC (rDLPFC) as previously succesfully applied by our research group (Jansen et al., 2015). For the sham intervention, stimulation parameters were identical, however, the coil was tilted 90° relative to the scalp. The rDLPFC was located at position F4 using the International 10–20 EEG system (Herwig et al., 2003). MT was determined at rest using single pulse TMS over the motor cortex. Stimulus intensity was adjusted until the muscular (left abductor pollicis brevis) response of the thumb muscular abduction was observed in five out of 10 stimuli. HF-rTMS treatment was applied using a 70 mm double air film coil (Magstim Co., United Kingdom) and a Magstim Rapid2 stimulator (Magstim Co., United Kingdom). The HF-rTMS treatment was added to the TAU provided by the Jellinek Addiction Treatment Centre in Amsterdam.

### Measures

#### Sample Characteristics

The following sample characteristics were assessed: age, gender (man/woman), IQ by means of the Dutch version of the adult reading test (NLV) (Schmand et al., 1991), years of education, handedness (left/right), MOCA score, presence of comorbid posttraumatic stress disorder (PTSD), cocaine or cannabis dependence by means of the Mini-International Neuropsychiatric Interview (MINI) (Sheenhan et al., 1998), duration of problematic alcohol use (years), number of DSM-IV criteria fulfilled (11 in total), use of anti-craving medication (naltrexone or acamprosate) (yes/no), use of anti-depressant medication at baseline (yes/no), use of sedative medication at baseline (yes/no), The Barratt Impulsiveness Scale (BIS) (Patton, 1995) total score and Urgency, Premeditation, Perseverance, Sensation Seeking (UPPS-P) impulsive behavior scale (Whiteside et al., 2005).

#### Impulsivity Assessments

##### Delay discounting task

**FIGURE 2:**
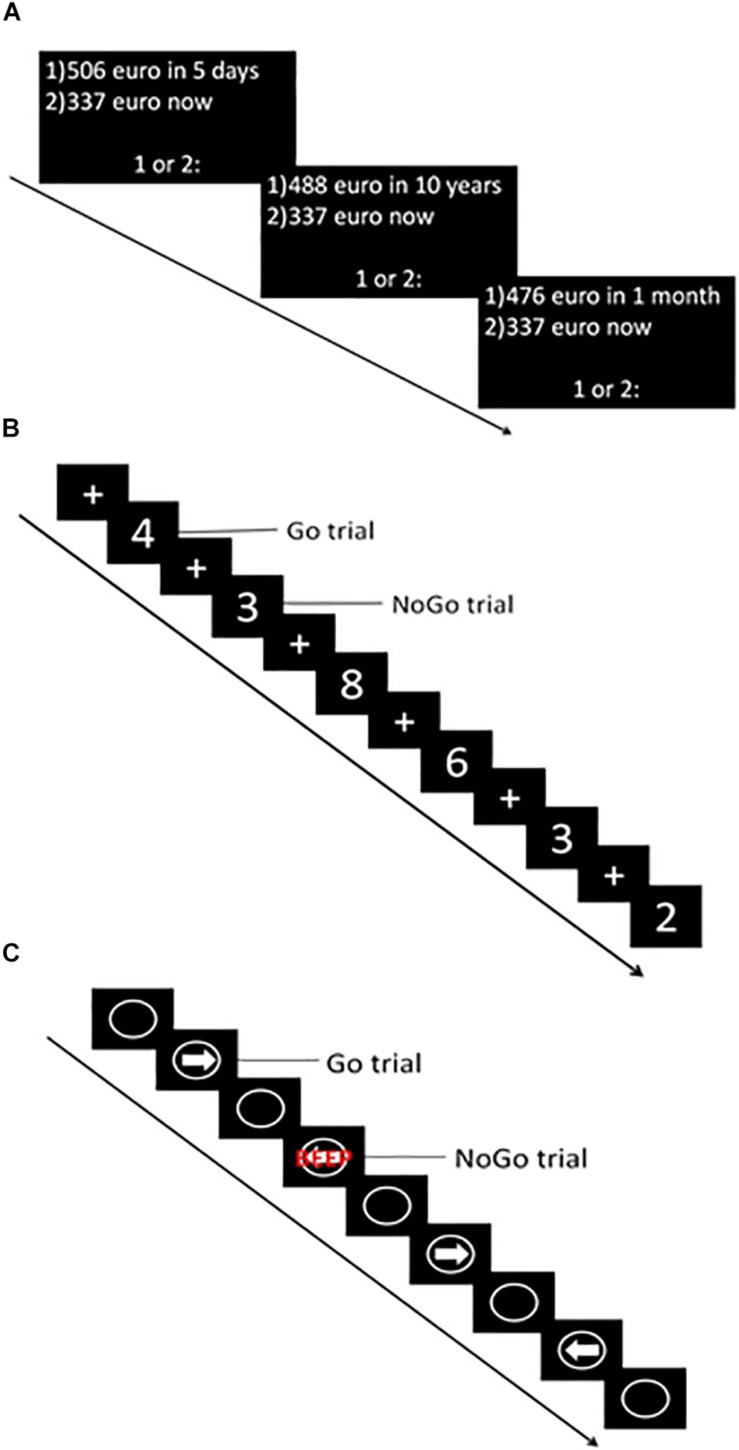
Graphical representation of impulsivity computer tasks. **(A)** Delay Discounting Task (DDT). **(B)** Go-NoGo Task (GNGT). **(C)** Stop Signal Task (SST).

The computerized version of the DDT (Wittmann et al., 2007) was used to assess choice impulsivity. During this task, participants were presented with a choice between a hypothetical smaller immediate or larger delayed reward. To choose the immediate reward option participants had to press the “c” key, while for the later reward participants had to press the “m” key on a keyboard. The task consisted of six blocks, each containing eight choices. Delay in days (i.e., 5, 30, 180, 365, 1095, 3650) and delayed reward in euros (ranging from 476 to 524 euros) were equal for all trials of a given block (Figure 2A). The immediate reward value varied across trials within each block, depending on the responses made. Within each block the indifference point (i.e., when the immediate reward has the same subjective value as the delayed reward) was determined. Using the normalized indifference points, a discounting curve was created for each participant. Subsequently, the area under the curve (AUC) (Myerson et al., 2001) was calculated with normalized delay (*x*-axis) and reward value (*y*-axis). The AUC was the primary outcome measure of this task. Lower values indicated higher choice impulsivity.

##### Go-nogo task

An adapted version of the GNGT (Durston et al., 2003) was used to assess failure to refrain from action initiation. During this task, white numbers (1–9) were projected in the middle of a black screen for 500 ms. Between the numbers, a fixation cross was projected for an average duration of 1500 ms (1000 ± 2000 ms). Participants were instructed to respond (press the spacebar of the keyboard) as fast as possible whenever a number (Go trial) was projected, but to refrain from responding when a “3” was projected (NoGo trial) (Figure 2B). The task consisted of five runs, each containing 57 trials (approximately 75% Go trials). During the entire task, 215 Go trials and 70 NoGo trials were presented. The primary outcome measure of this task was the percentage responses to NoGo trials (i.e., % false alarms) – with higher percentages indicating higher impulsivity. Furthermore, responses to Go trials (i.e., hits) and reaction time (RT) to Go trials were recorded.

##### Stop signal task

The CANTAB ([Cognitive assessment software]. Cambridge Cognition (2019). All rights reserved.^1^) version of the SST was used to assess the, inability to stop an initiated response. The task consisted of five blocks, each containing 64 trials. During each trial, a white circle was projected in the middle of a black screen wherein a white arrow pointing to the left or right appeared (Figure 2C). Participants were instructed to make a rapid response in the direction of the arrow [left button (F7) and right button (F8)] (Go trial). In some rare cases, the arrow was followed by an auditory beep (Stop trial), which indicated participants had to stop their initiated response and refrain from pressing the button. After each block, a feedback screen was displayed, which showed a blue bar representing the response time of the last block (each bar representing performance during one block). The higher the bar, the faster the participant responded during the last block. This was explained to the participant by the experimenter, and subsequently, the participant was encouraged to respond faster in the next block, but also to stop the initiated response when the stop signal was heard. In total, the task contained 240 go trials and 80 stop trials. During the stop trials, the time between stimulus presentation and stop signal in which a participant was able to stop in 50% of the trials was determined using a staircase procedure (with a successful inhibition the stop signal delay increased by 50 ms, whereas with failed inhibition it decreased with 50 ms). This time – referred to as the SSRT – was the primary outcome measure of this task, with higher numbers indicating higher impulsivity. Furthermore, the proportion of correct stops during stop trials and reaction time on go trials were recorded. All computerized tasks were performed on a manually operated touch screen tablet (Hewlett-Packard; Windows 8.1) with the keyboard attached.

#### Blinding

After the 10 HF-rTMS treatments, participants indicated whether they believed to have received the active or sham treatment.

#### Safety and Tolerability

In order to list the discomfort or side effects that participants experienced after treatment, a predetermined list of possible side effects was used. The list contained: headache, pain or beep in the ear, reduced hearing, fainting or epileptic seizure. In case a participant reported other side effects that were not on this list (uncomfortable sensations at stimulation site after the stimulation, and tiredness after stimulation), these side effects were registered as well.

### Analyses

Statistical Package For Social Sciences (SPSS) version 25.0 was used for analyses of sample characteristics, baseline differences in taks performance, blinding and safety and tolerability (IBM Corp., Released 2017. IBM SPSS Statistics for Windows, Version 25.0. Armonk, NY, United States: IBM Corp.). The R environment (RStudio Team (2015). RStudio: Integrated Development for R. RStudio, Inc., Boston, MA, United States) was used for statistical analyses of the treatment effect.

#### Sample Characteristics

Baseline sample characteristics were compared between the active and sham group. In case of a categorical variable, Chi-square tests were used (in case the expected counts is less than 5, Fisher’s exact test were used as an alternative). In case of a continuous variable, normality was tested by means of the Kolmogorov Smirnov test. A two-sample *T*-test was used in case of normality, otherwise the Mann–Whitney-*U* test was applied. *P*-values < 0.05 were considered significant.

#### Impulsivity

##### Determination of outliers

To determine whether participants performed the task according to instructions, specific criteria were set for each cognitive task. Participants determined as outlier were discarded from further data analysis for that specific task.

*DDT*: An outlier was defined based on non-systematic choice behavior and participants were excluded when: (1) at least one individual indifference point was greater than the preceding indifference point by a magnitude greater than 20% of the larger later reward; or (2) the last indifference point was not less than the first indifference point by at least a magnitude equal to 10% of the larger later reward (Johnson and Bickel, 2009).

*GNGT*: Outliers were determined based on the mean and standard deviation of RT on Go trials (three sessions combined). When a participant’s mean RT on Go trials exceeded this group mean by two times the standard deviation or more, the subject was considered an outlier.

*SST*: Outliers were based on the proportion of successful stops on NoGo trials (Bø et al., 2019). When the proportion of successful stops was lower than 0.4 or higher than 0.6 (indicating a failed staircase procedure), a participant was considered an outlier.

##### Baseline differences

To determine baseline differences between the active group and sham group, the primary outcome measures of the baseline session were tested for normality and accordingly compared using a two-sample *t*-test (normal distribution) or Mann–Whitney-*U* test (non-normal distribution). *P*-values < 0.05 were considered significant.

##### Treatment effect

The effect of treatment and session was determined using linear mixed-effects models. To check assumptions, the residuals of the primary outcome measures were visually inspected for normality using histograms and quantile-quantile-plots. The final model was selected by statistical (Chi-square) model comparison, assessing model fit using the Akaike Information Criterion (AIC) values, with lower values indicating better fit. The dependent variables were the primary outcome measures of the task, i.e., AUC for the DDT, false alarm percentage for the GNGT and SSRT for the SST. We started with a model including the fixed effects of treatment (active/sham) and session (pre/mid/post), as well as interaction term of treatment and session, and the random intercept of subject. Step-by-step extra fixed or random effects were added to this first model. The second model contained a fixed effect of age, whereas the third model contained a fixed effect of gender. The fourth model contained both age as well as gender as extra fixed effects. The fifth model added a random slope for session to the first model. The sixth model contained age as a fixed effect, the seventh model contained gender as a fixed effect and the eighth model contained age and gender as fixed effects. The AICs of all these models were compared by means of Chi-square tests. *P*-values < 0.05 were considered significant. The result of this test determined which model was chosen as final model for each specific task.

*DDT*: The final model included the fixed effects of session (pre/mid/post) and treatment (active/sham), as well as the interaction term of session and treatment and the random intercept of subject [AUC ∼ Session * Treatment_Group + (1 | Subject)]. Adding fixed effects of age [*X*^2^(1) = 0.106, *p* = 0.744], or age and gender [*X*^2^(1) = 0.102, *p* = 0.750], to this model did not significantly improve the model fit. Furthermore adding a random slope for session resulted in singular fit of the model (i.e., the variance- covariance matrix was estimated as zero), and was therefore not included in analyses of the AUC.

*GNGT*: The final model included fixed effects of session (pre/mid/post), treatment (active/sham), and age, as well as the interaction term of session and treatment, and a random intercept of subject and random slope for session [Percentage false alarms ∼ Session * Treatment Group + Age + (Session | Subject)]. Adding gender to this model did not significantly [*X*^2^(1) = 0.7518, *p* = 0.386] improve the model fit. This shows that gender does not explain variance, and was therefore not included in the final analyses of the false alarm percentage.

*SST*: The final model included fixed effects of session (pre/mid/post) and treatment (active/sham), as well as the interaction term of session and treatment, and a random intercept of subject and random slope for session [SSRT ∼ Session * Treatment Group + (Session | Subject)]. Adding age [*X*^2^(1) = 2.712, *p* = 0.0996], or age and gender [*X*^2^(2) = 2.809, *p* = 0.246] to this model did not significantly improve the model fit. This shows they do not explain any variance, and were therefore not included in the final analyses of the SSRT.

#### Exploratory Analyses

##### Baseline impulsivity

In order to assess whether baseline impulsivity (measured with the BIS and UPPS-P) had an effect on HF-rTMS treatment response, the baseline BIS, and the baseline UPPS-P score, were independently correlated (Pearson correlation) to the difference score (value session 10 – value session one) of the AUC (for the DDT), percentage false alarms (for the GNGT) and SSRT (for the SST) in the active group. Individuals that dropped out before the 10th session were discarded from these analyses. *P*-values < 0.05 were considered significant.

##### Severity of alcohol use disorder

To assess whether severity of AUD had an effect on HF-rTMS treatment response, the total number of DSM-IV criteria met was correlated (Pearson correlation) to the difference score (value session 10 – value session one) of the AUC (for the DDT), percentage false alarms (for the GNGT) and SSRT (for the SST) in the active group. Individuals that dropped out before the 10th session were discarded from these analyses. *P*-values < 0.05 were considered significant.

#### Blinding

In order to assess whether blinding succeeded, the percentage of individuals who guessed their treatment allocation correctly were calculated. Subsequently, a binomial test was used to determine whether this was significantly different from chance level (50%). *P*-value < 0.05 was considered significant.

#### Safety and Tolerability

Chi-square tests, and Fisher’s exact tests were performed to assess whether there were any statistical differences in reported discomfort or side effects between the active group and the sham group. A *p*-value < 0.05 was considered significant.

## Results

### Sample Characteristics

**FIGURE 3:**
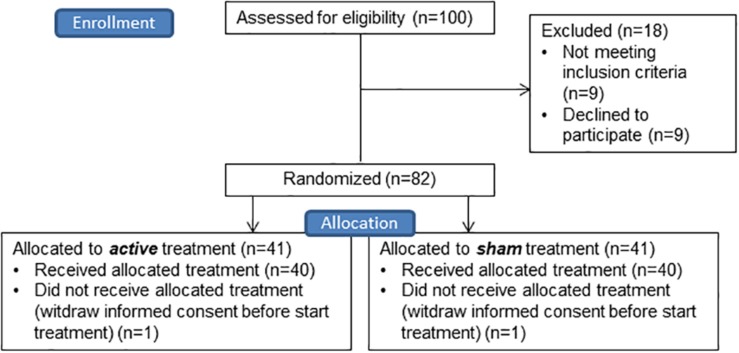
CONSORT flow diagram of the enrollment and allocation phase of the study. For drop out during analyses per task see Figure 4.

In total, hundred individuals were screened, which resulted in 82 enrolled individuals. Two participants withdrew informed consent before the procedure started, therefore in total eighty participants started the study (see Figure 3). No significant differences in age, gender, IQ, years of education, handedness, MOCA score, presence of comorbid PTSD, cocaine or cannabis dependence, duration of problematic alcohol use, number of DSM-IV criteria fulfilled, anti-craving medication use at baseline, use of sedative medication, BIS total score and UPPS total score between the active and sham group were found. Use of anti-depressive medication did significantly differ between the sham and active group, with more use in the active group [*X*^2^(1) = 4.013, *p* = 0.045] (see Table 1).

**TABLE 1:** Sample characteristics.

	**Active group (*N* = 40)**	**Sham group (*N* = 40)**	**Statistic**
Age [mean (SD)]	44.95 (10.03)	43.75 (11.41)	*t*(78) = 1.498, *p* = 0.619
Gender (man: woman)	29: 11	31: 9	*X*^2^(1) = 0.267, *p* = 0.606
IQ [median (range)]	83 (47–97)	84 (42–100)	*U* = 772.500, *p* = 0.791
Years of education [mean (SD)]	7.5 (3.44)	7.238 (3.61)	*t*(78) = 0.333, *p* = 0.740
Handedness (right: left)	37: 3	38: 2	*p* = 1.000, Fisher’s exact test
MoCA score (>27: 18–26)	26: 13	30: 10	*X*^2^(1) = 0.664, *p* = 0.415
PTSD (yes: no)	5: 35	6: 34	*X*^2^(1) = 0.105, *p* = 0.745
Cocaine dependence (yes: no)	9: 31	5: 35	*X*^2^(1) = 1.385, *p* = 0.239
Cannabis dependence (yes: no)	8: 32	8: 32	*X*^2^(1) = 0.000, *p* = 1.00
Duration of problematic alcohol use in years [mean (range)]	11 (2–36)	10 (1–36)	*U* = 652.500, *p* = 0.211
Number of DSM-IV criteria fulfilled [median (range)]	9 (3–11)	9 (4–11)	*U* = 775.000, *p* = 0.808
Anti-craving medication (yes: no)	15: 25	12: 28	*X*^2^(1) = 0.503, *p* = 0.478
Anti-depressant medication (yes: no)	15: 25	7: 33	*X*^2^(1) = 4.013, *p* = 0.045
Sedative medication (yes: no)	3: 37	1: 39	*p* = 0.615, Fisher’s exact test
BIS score [mean (SD)]	69.53 (8.72)	69.58 (9.42)	*t*(78) = −0.025, *p* = 0.980
UPPS score [mean (SD)]	108.60 (14.40)	110.65 (16.00)	*t*(78) = −0.602, *p* = 0.549

*No significant differences were found between the active and sham group on any of the characteristics, except for the use of anti-depressant medication. Depending on whether the outcome measure was continuous or categorical, and whether it was normally distributed, two sample *T*-test, Mann–Whitney-*U* test, Chi–square tests or Fisher’s exact test were used. *P*-values < 0.05 were considered significant. Abbreviations: SD = standard deviation; PTSD = post-traumatic stress disorder; DSM-IV: diagnostic and statistical manual of mental disorders version 4; BIS = barratt impulsiveness scale; UPPS = urgency, premeditation, perseverance, sensation seeking impulsive behavior scale.*

**FIGURE 4:**
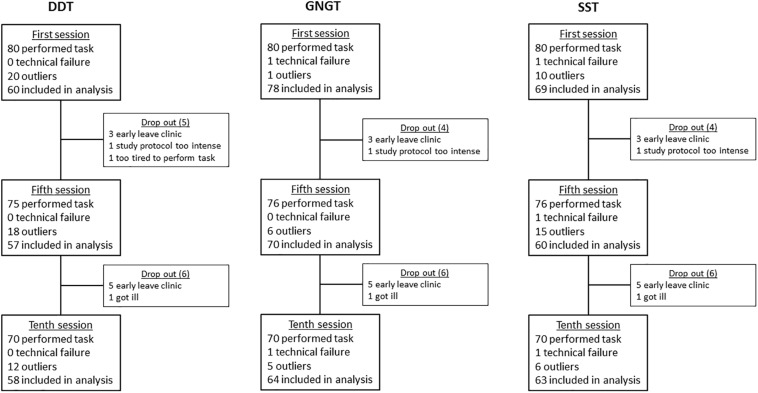
Schematic overview of drop out, data loss, and outliers of the Delay Discounting Task (DDT), Go-NoGo Task (GNGT), and Stop Signal Task (SST).

### Impulsivity

#### Data Loss per Task

For a schematic overview of drop-outs, data loss, and outliers per task see Figure 3.

#### Baseline Differences

*DDT*: The two sample *t*-test showed no significant difference in mean AUC between the active [0.40 (0.214)] and sham [0.50 (0.279)] group [*t*(58) = −1.509, *p* = 0.137].

*GNGT*: The Mann–Whitney-*U* test showed no significant difference in false alarm percentage between the sham [28.571% (11.429–84.286%)] and active [27.143% (2.857–75.714%] treatment group (*U* = 864.500, *p* = 0.298).

*SST*: The two sample *t*-test showed no significant difference between the mean SSRT of the active [190.75 ms (46.383 ms)] and sham [188.61 ms (54.688 ms)] group [*t*(67) = 0.174, *p* = 0.862].

#### Treatment Effect

**FIGURE 5:**
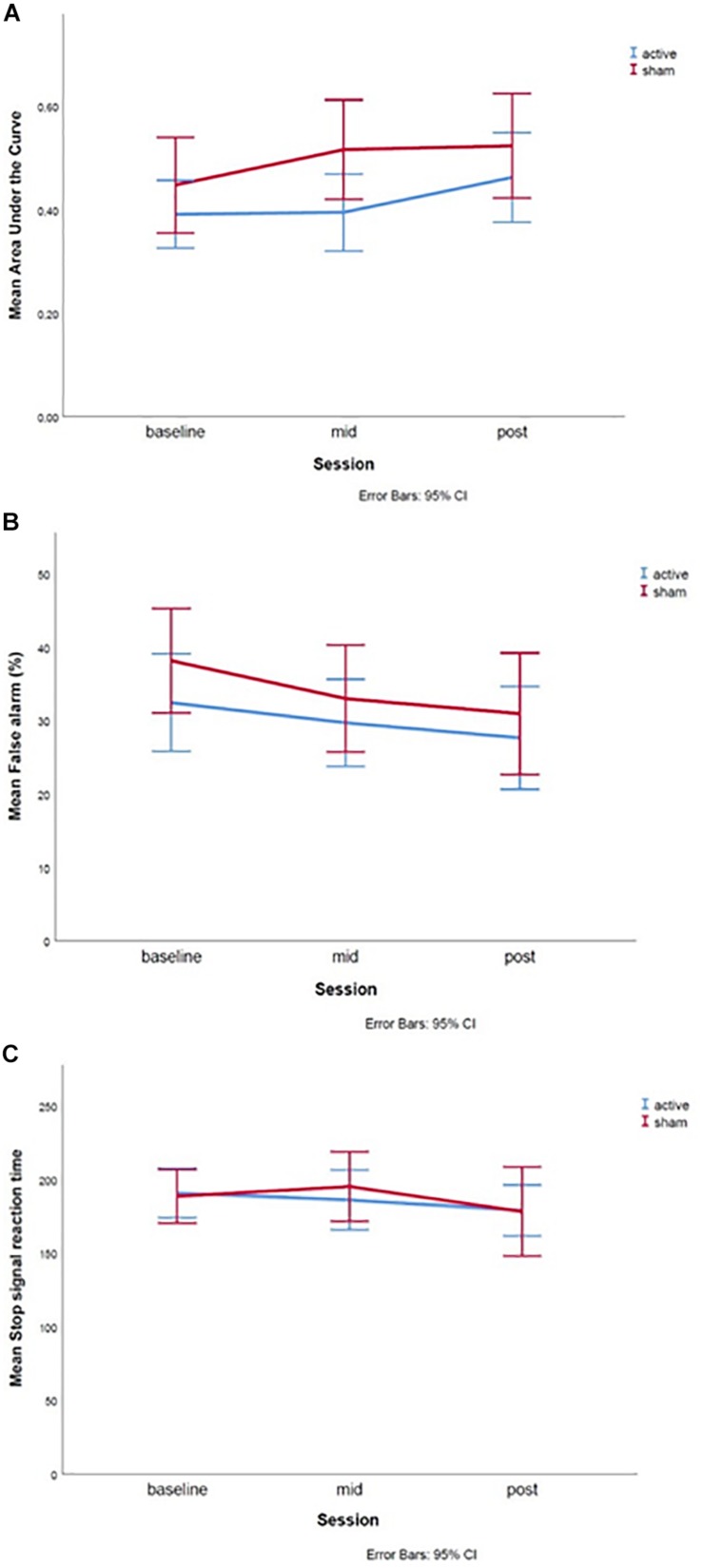
Graphs of the treatment effect for the **(A)** Delay Discounting Task, **(B)** Go-Nogo Task, and **(C)** Stop Signal Task. The baseline measures were assessed during the first test day, mid during the fifth test day and post during the 10th test day. Error bars represent 95% confidence intervals.

*DDT*: The linear mixed-effects model showed no significant main effects of session [*T*(102.30) = 0.910, *p* = 0.365], or treatment group [*T*(120.50) = 1.006, *p* = 0.317], nor an interaction effect between session and treatment [*T*(102.2) = 0.025, *p* = 0.980] was found (Figure 5A).

*GNGT*: The linear mixed-effects model showed no significant main effects of session [*T*(69.260) = −0.364, *p* = 0.717], treatment group [*T*(73.919) = 0.936, *p* = 0.353] or an interaction effect between session and treatment [*T*(68.267) = −0.468, *p* = 0.642] was found (Figure 5B). However, the fixed effect of age was significant [*T*(72.446) = −2.004, *p* = 0.049], such that higher age was related to lower percentage of false alarms.

*SST*: The linear mixed-effects model showed no significant main effects of session [*T*(73.745) = −0.653, *p* = 0.516], or treatment group [*T*(76.160) = −0.228, *p* = 0.820], nor an interaction effect between session and treatment [*T*(71.870) = 0.371, *p* = 0.712] was found (Figure 5C).

#### Exploratory Analyses

##### Baseline impulsivity

*DDT*: The Pearson correlation revealed no significant relationship between UPPS-P score (*r* = −0,131, *n* = 24, *p* = 0,543) or BIS score (*r* = −0.154, *n* = 24, *p* = 0.472) and AUC difference score.

*GNGT*: No significant correlation was found between the UPPS-P score (*r* = −0.064, *n* = 30, *p* = 0.738) or BIS score (*r* = 0.128, *n* = 30, *p* = 0.502) and the difference score of false alarm percentage.

*SST*: A correlation trending significance was found between the UPPS-P score and the SSRT difference score (*r* = 0.349, *n* = 25, *p* = 0.088). However, this effect was driven by one participant with a high difference score and high UPPS-P score. When this participant was removed from analyses the correlation decreased (*r* = 0.100, *n* = 24, *p* = 0.641). No significant correlation was found between BIS score and SSRT difference score (*r* = −0.032, *n* = 25, *p* = 0,880).

##### Severity of alcohol use disorder

*DDT*: Pearson correlation did not reveal a significant relationship (*r* = −0.174, *n* = 24, *p* = 0.417) between severity of AUD and AUC difference score.

*GNGT*: No significant correlation (*r* = 0.147, *n* = 30, *p* = 0.439) was found between the severity of AUD and the difference score of percentage false alarms.

*SST*: Severity of AUD and SSRT difference score also did not significantly correlate (*r* = 0.193, *n* = 25, *p* = 0.356).

#### Blinding

Data on treatment allocation was collected from 68 participants. 39 individuals believed to have received active treatment while 29 believed to have received sham treatment. 63.24% of the participants guessed their treatment allocation accurately. The binomial test indicated that the observed proportion of individuals who guessed their treatment allocation correctly (0.63) is significantly higher than the expected chance level (0.50) (*p* = 0.038).

#### Safety and Tolerability

In the active group in total 372 stimulation sessions were applied. Headache after stimulation occurred seven times (1.9%), pain or beep in the ear occurred three times (0.8%), tiredness after stimulation occurred two times (0.54%) and unpleasant sensation at stimulation site after stimulation occurred nine times (2.4%). In the sham group in total 366 stimulation sessions were applied. The same side effects were reported: headache occurred 17 times (4.6%), tiredness after stimulation occurred two times (0.55%) and unpleasant sensations at stimulation site occurred two times (0.55%). No pain or beep in the ear was reported in the sham group. The active group experienced significantly less headache compared to the sham group [*X*^2^(1) = 4.477, *p* = 0.034]. However, the sham group experienced significantly less unpleasant sensations at the stimulation site after stimulation [*X*^2^(1) = 4.407, *p* = 0.036], compared to the active group. Groups did not differ on the other reported side effects [pain or beep in the ear (*p* = 0.249, Fisher’s exact test)/tiredness after stimulation (*p* = 1.000, Fisher’s exact test)].

## Discussion

The current study aimed to elucidate the effect of 10 HF-rTMS sessions on impulsivity measures in abstinent individuals in treatment for AUD. The add-on HF-rTMS treatment was tolerated well, since no severe side effects were reported. Impulsivity was assessed by the Delay Discounting, Go-NoGo and SSTs that were performed before, midway, and post HF-rTMS treatment. Contrary to the hypotheses, the current results suggest no effect of 10 HF-rTMS sessions on performance on any of the impulsivity tasks.

To the best of our knowledge, this study was the first to assess the effect of 10 sessions of HF-rTMS treatment in AUD on impulsivity, as measured by three impulsivity tasks. The SST was never before used to study improvements of impulsivity in alcohol (or substance) dependence using HF-rTMS, however, impulsivity, using the GNGT and DDT, was studied in this population. The lack of an effect on accuracy on the GNGT in the current study is in line with the study of Herremans et al. (2013), who also did not find an effect of one session of HF-rTMS treatment on GNGT accuracy in alcohol dependent patients. Contrarily, however, a sham-controlled study of Del Felice et al. (2016), in which four sessions of HF-rTMS treatment were applied, did find increased accuracy on the GNGT in AUD patients. Furthermore, the current results are contrary to the study of Sheffer et al. (2013) who report decreased discounting as measured by the DDT after one single session of HF-rTMS in nicotine dependent patients. The discrepancy is unexpected in light of the number of stimulation sessions, since applying multiple sessions of HF-rTMS could induce summation of the effect of a single session (Valero-Cabré et al., 2008) and therefore could be expected to have a larger effect. This was, however, not confirmed in the current study since we applied 10 sessions and did not find an effect on impulsivity measures. An explanation for this inconsistency might be the difference in the clinical status of the treated individuals. In the current study, severe AUD patients in treatment with an intention to quit were included, whereas Sheffer et al. (2013) treated nicotine dependent patients who did not have the intention to quit smoking. Individuals suffering from alcohol or marijuana dependence are more prone to facing social and economic problems in society (Cerdá et al., 2016). Hence, one may argue that worse clinical status requires more stimulation sessions in order to achieve an effect. However, this is not in line with the studies of [opetwcite]B17,B10[clotwcite]Herremans et al. (2013); Del Felice et al. (2016) and the current study, since these studies included clinical groups, but have different results. Altogether, this suggests that results of HF-rTMS studies in alcohol and substance use disorders interfering with impulsivity are still mixed and inconclusive.

The current study did find a significant effect of age on GNGT performance: older individuals made less false alarms, indicative of decreased impulsivity. This is in line with Steinberg et al. (2008), who report a negative association between age and impulsivity. For the GNGT specifically, it has been found that with increasing age performance improves, however, when individuals reach older adulthood, performance decreases again (Votruba and Langenecker, 2013). However, these studies were performed in a sample of non-clinical individuals. Although, several studies address impulsivity in different age categories in substance use disorder (Argyriou et al., 2018), the relationship between age and impulsivity task performance in AUD has not been studied directly. However, it should be noted that we only find the effect of age for the GNGT, which assesses the failure to refrain from action initiation. Choice impulsivity (as measured with the DDT) and failure to inhibit an already initiated response (as measured with the SST) were not affected by age in the current study. Whether age only has a positive effect on action initiation is a topic for future research.

Some variability in inter-individual factors might contribute to the effect of non-invasive neuromodulation (Li et al., 2015) on certain outcome measures. Although deriving from transcranial Direct Current Stimulation studies, these inter-individual factors might also hold for HF-rTMS. Suggested inter-individual factors are: baseline performance, severity of disorder, age and gender. We performed several analyses in order to see whether the null results of the current study changed when these factors were taken into account. To begin with, baseline impulsivity measures as well as severity of AUD, did not affect the effect of HF-rTMS treatment on cognitive measures. Moreover, no significant effects of age and gender on the effect of HF-rTMS on impulsivity measures was found. Therefore current null findings cannot be explained by these factors. Other factors that might contribute to the effectiveness of non-invasive neuromodulation, and which were not directly measured in the current study are: anatomy, functional organization of local circuits, task related neurophysiology, neurochemistry and genetics (Li et al., 2015). Future studies should address whether these factors also influence effect of HF-rTMS in AUD. However, it is debatable whether trials studying the clinical application of HF-rTMS in psychiatry are suitable for these – more fundamental – neurobiological factors. Finally, the effect of HF-rTMS might depend on the choice of stimulation parameters. To begin with, one might argue that longer stimulation (more TMS pulses per session) induces stronger effects (Schulze et al., 2018). Furthermore, the number of repetitions, with the perfect interval, also influences the effect. However, studies systematically comparing different repetition schemes are currently missing (Ekhtiari et al., 2019). Additionally, the stimulation intensity might also influence the effect of stimulation (Lefaucheur et al., 2014). Altogether, future research must determine whether there are optimal settings for treatment of AUD (Ekhtiari et al., 2019).

The current study is the first randomized controlled clinical trial to apply 10 sessions of HF-rTMS over the right DLPFC to eighty AUD patients in treatment. One limitation of the current study was the sham condition. During sham treatment, the coil was tilted away 90 degrees from the scalp, which caused the magnetic field to flow away instead of passing the skull. The downside of this type of sham stimulation is that sensations on the scalp are also eliminated. Moreover, the participants of the current study were able to communicate with each other about the physical sensations they were experiencing during the HF-rTMS treatment since they were all admitted at the same department of the addiction treatment center. In line, participants guessed their treatment allocation correctly slightly above chance level. Therefore, future studies with patients that are hospitalized at the same department are recommended to consider using alternative types of sham stimulation, for example, a specific sham coil that mimics the sensations on the scalp (Rossi et al., 2007). Furthermore, a recent study in AUD patients (Moritz et al., 2018) indicated that other factors, such as motivation and effort may influence neurocognitive task performance in AUD to a larger extent than in healthy controls. In this study, motivation and effort partially mediated the diminished neurocognitive performance in AUD patients, which may also result in larger variability in between test sessions. In our study, large variability in the impulsivity measures was present, indicating high variability in impulsivity within the AUD patients, which could partially be explained by motivation. The variability between the test sessions may have resulted in diminished sensitivity to find group by test session interactions. It is therefore recommended to include measures of motivation and effort in neurocognitive test batteries in substance use disorder studies (Moritz et al., 2018), to determine whether high variability in the outcome measures is caused by motivation.

The current study was one of the first assessing the effect of HF-rTMS treatment on impulsivity in patients with AUD in clinical treatment. Results indicated no additional effect of this treatment on impulsivity measures. Future studies are required to investigate whether blinding with a sham coil would affect results and whether impulsivity declines with age in AUD patients.

## Data Availability Statement

The datasets generated for this study are available on request to the corresponding author.

## Ethics Statement

The studies involving human participants were reviewed and approved by the Medical Ethical Committee of the Academic Medical Centre Amsterdam (2015_064). The patients/participants provided their written informed consent to participate in this study.

## Author Contributions

RS, RH, and AG contributed to the design of the study. RS performed the study and analysis. RS wrote the first draft of the manuscript. RH and AG revised the manuscript. All authors read and approved the final version of the manuscript.

## Conflict of Interest

The authors declare that the research was conducted in the absence of any commercial or financial relationships that could be construed as a potential conflict of interest.

## Footnotes

1 www.cantab.com
